# The potency of the broad spectrum bacteriocin, bactofencin A, against staphylococci is highly dependent on primary structure, N-terminal charge and disulphide formation

**DOI:** 10.1038/s41598-018-30271-6

**Published:** 2018-08-07

**Authors:** Paula M. O’ Connor, Eileen F. O’ Shea, Paul D. Cotter, Colin Hill, R. Paul Ross

**Affiliations:** 1 Teagasc Food Research Centre, Moorepark, Fermoy, Co. Cork Ireland; 2 APC Microbiome Ireland, University College Cork, Cork, Ireland; 3 School of Microbiology, University College Cork, Cork, Ireland

## Abstract

Bactofencin A is a novel class IId bacteriocin, produced by the intestinal isolate *Lactobacillus salivarius* DPC6502, which has potent activity against medically significant pathogens including *Staphylococcus aureus*. This bacteriocin is unusual in that it has a highly cationic N terminus and a single disulfide bond between Cys7 and Cys22, resulting in a large C terminal loop. In this study, a library of synthetic bactofencin A variants were screened against the mastitis isolate, *S*. *aureus* DPC5246, to identify key residues responsible for activity. It was apparent that substituting either cysteine of the disulfide bond with either serine or alanine significantly reduced the activity of the bacteriocin, confirming the importance of the C terminal loop. Substituting N terminal amino acids with alanine had no effect on activity, whereas sequential removal of the N terminal positively charged residues resulted in an increasingly inactive peptide. A complete (synthetic) alanine scanning analysis revealed that the residues between Val9 and Gly17 were most affected by substitution suggesting that this area has a major influence on the potency of the bacteriocin. Substituting residues in the loop region between Cys7 and Cys22 for D-amino acid equivalents had a more detrimental effect on activity than L-alanine substitutions. Specifically Y10A, N11A, P15A and T16A are active at 4, 16, 1 and 16 μM respectively while their D equivalents were inactive at 1000 μM, the highest concentration tested. Ultimately, this study identifies the critical features in the primary structure of the bacteriocin which gives it such potent activity against pathogenic staphylococci.

## Introduction

Concerns about the increased incidence of antimicrobial resistance (AMR) against human pathogens have led to calls for global efforts to combat this worrying phenomenon. If left untackled once treatable infections will again become incurable^1^. The Global Antimicrobial Resistance Surveillance System (GLASS) was set up in 2015 to standardise the collection and sharing of data on AMR at a global level and to promote coordinated action. In support of this initiative the WHO recently surveyed the development of new antibiotics in the clinical pipeline against priority pathogens and found that there is particular need for new classes of antimicrobial to abate the threat of AMR. Specifically, they found that most antibiotics are derived from existing antibiotic classes and these are only considered a temporary solution to AMR as they will be quickly rendered ineffective by existing resistance mechanisms. Expressly they state that more investment is needed in fundamental drug discovery to discover more innovative antimicrobials against priority pathogens including *Mycobacterium tuberculosis*, *Clostridium difficile* and *Staphylococcus aureus*^2^.

One class of antimicrobial that is receiving increased attention is the bacteriocins. These are stable peptides naturally produced by many bacteria and have potent activity against other bacteria including antimicrobial resistant pathogens^3^. Bacteriocin production by intestinal strains is considered a desirable probiotic trait that could potentially mediate an effect in three different ways; it may allow the producing strain to compete in the crowded gut ecosystem, it could provide protection to the host against pathogens and could potentially signal the immune system in a similar fashion to host antimicrobial peptides (AMPs)^4–7^.

Recently, we described bactofencin A, a small, positively charged bacteriocin produced by the porcine gut isolate *L*. *salivarius* DPC6502. Structurally, it consists of a positively charged N terminal attached to a C terminal loop formed via a disulfide bond between Cys7 and Cys22. Bactofencin A is highly cationic and has been compared to eukaryotic defensins which contain a high Lys/Arg molar ratio considered essential for bactericidal activity^8,9^. Another unusual feature of bactofencin A is that immunity is mediated through a *dltB* homologue proposed to decrease the negative charge of the cell wall thereby reducing attraction between bacteriocin and cell, rather than a specific immunity protein. Bactofencin A displays activity against *S*. *aureus* and *Listeria* spp. and has been shown to subtly modify gut populations^10,11^.

The rational design of novel antimicrobials is rapidly evolving *via* the use of bioengineering to generate novel bacteriocin variants with enhanced functionality. This has been realised through the recent generation of both one and two peptide bacteriocins with greater activity against food-borne and medically significant Gram-positive and Gram-negative pathogens^12–17^. Indeed, nisin V, a single Met21Val substitution variant of the well characterised commercial bacteriocin nisin A generated in our laboratory, has greater *in vivo* efficacy against *Listeria monocytogenes* when compared with the native peptide^18^. In many cases, the identification of enhanced derivatives has been realised following initial studies in which saturation or scanning mutagenesis have been employed to reveal key important residues and structures within the peptide^12^.

The aim of this study was to determine the importance of specific residues and regions within bactofencin A to its anti-*S*. *aureus* activity. Bactofencin A is a relatively short Class IId peptide in which a disulfide bond naturally forms, making it especially amenable to peptide synthesis as a means of carrying out such structure-function investigations. Specifically a saturation approach was undertaken as follows; firstly, both cysteines were substituted with serine and alanine, individually and in tandem, to elucidate their role in peptide structure. Secondly, deleted variants were synthesised to assess the function of the positively charged N terminal. Thirdly, alanine was substituted for each amino acid (alanine scanning) and finally, a series of D-amino acid variants specific to the loop were synthesised to determine if chiral interactions with a receptor were likely to be involved in activity.

## Materials and Methods

### Peptide synthesis

Bactofencin A and variants were synthesised from the C terminus to the N terminus using microwave-assisted solid phase peptide synthesis (MW-SPPS) on a Liberty Blue microwave peptide synthesizer (CEM Corporation. Mathews, North Carolina, USA). Peptides with a C terminal cysteine were synthesized on an H-Cys(Trt)-HMBP pre-loaded resin, bactofencin C22A on an H-Ala-HMBP resin and bactofencin C22Cd on HMBP resin where the initial D-Cys was manually added to the resin (PCAS BioMatrix Inc., Quebec, Canada). The amino acid attached to the resin and the following two amino acids were deprotected conventionally at 25 °C, 0 W for 900 seconds in 5% piperizine in DMF to limit the formation of an undesirable 51 Da modification commonly seen in C terminal cysteine peptides. Following deprotection the exposed amino group is coupled conventionally with the carbonyl group of the next amino-protected amino acid at 75 °C, 0 W for 3600 seconds in the presence of the activator, 0.5 M N,N′-diisopropylcarbodiimide in DMF, and activator base, 1.1 M hydroxybenzotriazole in DMF. The fourth and subsequent amino acids were added using microwave deprotection at 75 °C, 60 W for 600 seconds and microwave coupling at 75 °C, 35 W for 600 seconds. Arginines were double coupled at 75 °C, 35 W, 300 seconds and histidine and cysteine coupled at 25 °C, 0 W, 300 seconds and then 50 °C, 35 W for 900 seconds. Following synthesis, the peptide was cleaved from the resin by adding a cleavage mix containing 9.25 ml trifluoroacetic acid (TFA), 250 µl water, 250 µl 2′2-(ethylenedioxy)-diethanethiol and 500 µl triisopropylsilane. This mixture was then heated at 37 °C for 1 hour to cleave the peptide from the resin. Resin was removed from the cleavage mix using an Accent Cleavage system (CEM, Corporation. Mathews, North Carolina, USA) and the TFA evaporated by bubbling with nitrogen. Peptide was precipitated from the remaining solution by adding 45 ml of diethyl ether pre-cooled to −20 °C and centrifuging at 1000 g for 3 minutes. The precipitated peptide was washed free of scavengers by resuspending in a second aliquot of 45 ml ice cold diethyl ether and the centrifugation step repeated.

### Purification of synthetic peptides

Crude peptide was purified using Reversed Phase-HPLC on a semi preparative Jupiter Proteo (10 × 250 mm, 4 µ, 90 Å) column (Phenomenex, Cheshire, UK) running an 11–45% acetonitrile 0.1% TFA gradient over 40 minutes where buffer A is Milli Q water containing 0.1% TFA and buffer B is 90% acetonitrile containing 0.1% TFA. Fractions with the desired molecular mass were identified using matrix assisted laser deionisation -time of flight-mass spectrometry (MALDI-TOF-MS) on an Axima TOF^2^ MALDI TOF mass spectrometer (Shimadzu Biotech, Manchester, UK) operating in positive ion reflectron mode and were pooled and lyophilized on a Genevac HT 4X lyophilizer (Genevac Ltd., Ipswich, UK). Peptides were resuspended in 50 mM sodium phosphate buffer pH 6.8 at approximately 1000 µM and kept at room temperature for 24–48 hours until the disulfide bond between Cys7 and Cys22 fully formed as monitored by MALDI-TOF-MS. Peptides were then further purified by a second HPLC run as described above except the gradient used was 15–30% acetonitrile 0.1% TFA gradient over 30 minutes. Again fractions containing pure bactofencin A were identified and lyophilised for specific activity experiments.

### Purification of natural bactofencin A

Bactofencin A was purified from MRS culture media as described by O’Shea *et al*.^10^. Briefly peptide was purified from an overnight culture of *L*. *salivarius* grown in 1 litre of MRS media using SP sepharose Cation Exchange, C18 Solid Phase Extraction (SPE) and Reversed Phase HPLC.

### Specific activity of bactofencin A variants

Peptides were resuspended at 1000 µM in 50 mM sodium phosphate buffer pH 6.8 and checked for purity by MALDI-TOF-MS. Peptides were serially diluted 1 in 4 to give a dilution series of 250, 62.50, 15.63, 3.91, 0.98 and 0.24 µM and assayed by the agar well diffusion assay described by Ryan *et al*.^19^. Briefly, 50 µl aliquots of each peptide concentration were plated on a *S*. *aureus* DPC5246 indicator plate and the plate incubated at 37 °C. Peptide activity (Minimum Inhibitory Concentration (MIC)) was taken as the lowest concentration of peptide to give a zone of inhibition. All assays were performed in triplicate. The dilution series values were rounded to the nearest whole number to give 1000, 250, 63, 16, 4, 1 and 0.25 µM and data is colour coded for ease of interpretation.

### Bactofencin variants synthesised for specific activity studies

Bactofencin A, KRKKHRCRVYNNGMPTGMYRWC, is a 22 amino acid bacteriocin with a disulfide bond between Cys7 and Cys22. Bactofencin variants are labelled according to the amino acid position number using the one letter code followed by the change it undergoes e.g. a lysine at position 1 to alanine change is labelled K1A.

Bactofencin Cys7 and Cys22 were substituted with serine both individually and in tandem to give bactofencin C7S, bactofencin C22S and bactofencin C7S-C22S. These amino acids were also substituted with alanine to give bactofencin C7A, bactofencin C22A and bactofencin C7A-C22A.

To assess the importance of the positively charged N terminal the following deletion variants were synthesised; bactofencin R2-C22, K3-C22, K4-C22, H5-C22, R6-C22 and C7-C22.

Each amino acid in bactofencin A was changed to alanine to give a library of alanine scanning variants, these are specifically K1A, R2A, K3A, K4A, H5A, R6A, C7A, R8A, V9A, Y10A, N11A, N12A, G13A, M14A, P15A, T16A, G17A, M18A, Y19A, R20A, W21A and C22A. Bactofencin C7A and C22A were also used to assess the role of cysteine in activity as described above.

Each amino acid in the loop from Cys7 to Cys22 was substituted for a D-amino acid equivalent to give a series of variants namely bactofencin C7Cd, R8Rd, V9Vd, Y10Yd, N11Nd, N12Nd, M14Md, P15Pd, T16Td, M18Md, Y19Yd, R20Rd, W21Wd and C22Cd. An all D-amino acid variant for a preliminary MIC_50_ assay was synthesised by Alta Bioscience (Birmingham, UK).

### Comparison of bactofencin A and bactofencin R8Q

Bactofencin R8Q and bactofencin R8K were synthesised as described above and their activity compared to bactofencin A against *S*. *aureus* DPC5246, *Listeria innocua* DPC3572 and *L*. *monocytogenes* ATCC 23074.

## Results

### Formation of the disulfide bond in synthetic bactofencin A

To investigate how specific residues and domains within bactofencin A contribute to its potency against staphylococci, a series of bactofencin variants were synthesized, purified and assayed. Natural bactofencin A is encoded on a four gene operon that includes an accessory protein that ensures the correct formation of the disulfide bond^10^. Although synthetic peptides are initially synthesized without a disulfide bond, this bond appears to form naturally given that we can detect it by MALDI TOF MS. Synthetic bactofencin A (2784 Da) in the reduced form was resuspended in sodium phosphate buffer pH 6.8 at 1000 µM, and bond formation (i.e. presence of peptide at 2782 Da) monitored over time by MALDI TOF MS analysis (data not shown). For subsequent investigations, synthetic peptides were HPLC purified, resuspended in sodium phosphate buffer until disulfide bond formation occurred (where appropriate), as confirmed by MALDI TOF MS, and then HPLC purified for a second time to obtain pure peptide with an intact disulfide bond.

### Comparison of natural and synthetic bactofencin A

**Figure 1:**
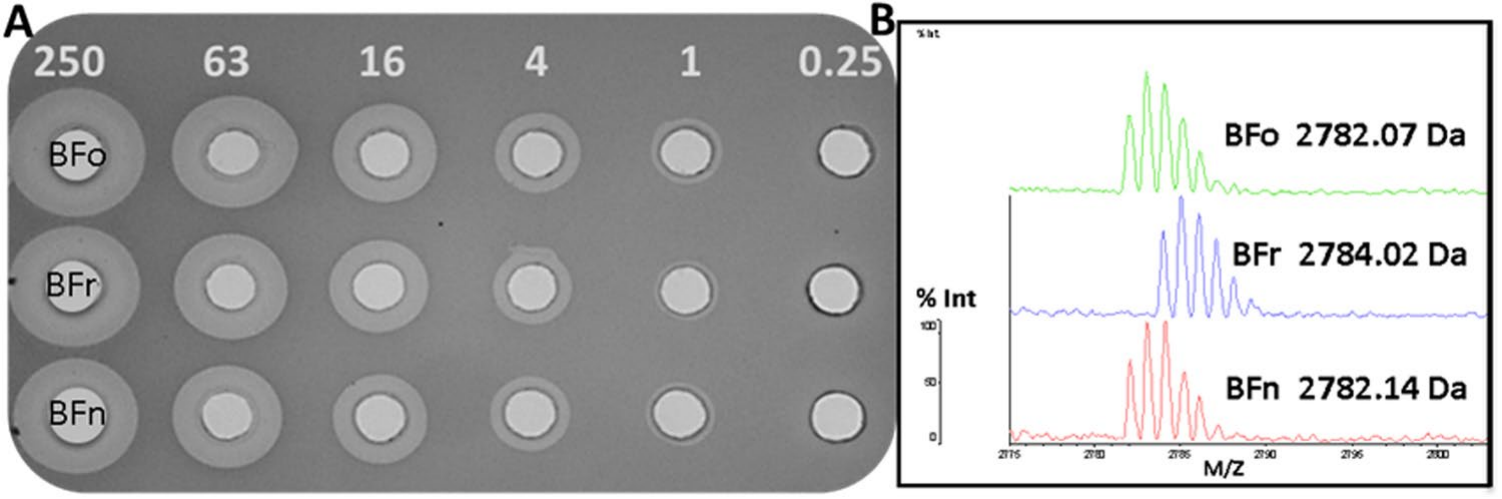
Activity (µM) of oxidized synthetic bactofencin A (BFo), reduced bactofencin A (BFr) and natural bactofencin A (BFn) against *S*. *aureus* DPC5246 (**A**) and molecular mass of oxidized synthetic bactofencin A (Top), reduced bactofencin A (Middle) and natural bactofencin A (Bottom) (**B**).

The yield of natural bactofencin A following purification from *L*. *salivarius* DPC6502 culture media is generally very low (<0.3 mg/L) making it difficult to generate sufficient peptide for structure/function experiments and making genetic approaches to generating peptide variants impractical. Bactofencin A, being a small 22 amino acid peptide with a single disulfide, is well suited to peptide synthesis and this approach generated milligram quantities of peptide variants for specific activity studies. Comparison of activity of natural bactofencin A (2782 Da) with synthetic reduced bactofencin A (2784 Da) and synthetic oxidised bactofencin A (2782 Da) showed that all peptides were equally active against *S*. *aureus* DPC5246 (Fig. 1).

### The role of cysteines in bactofencin A

**Figure 2:**
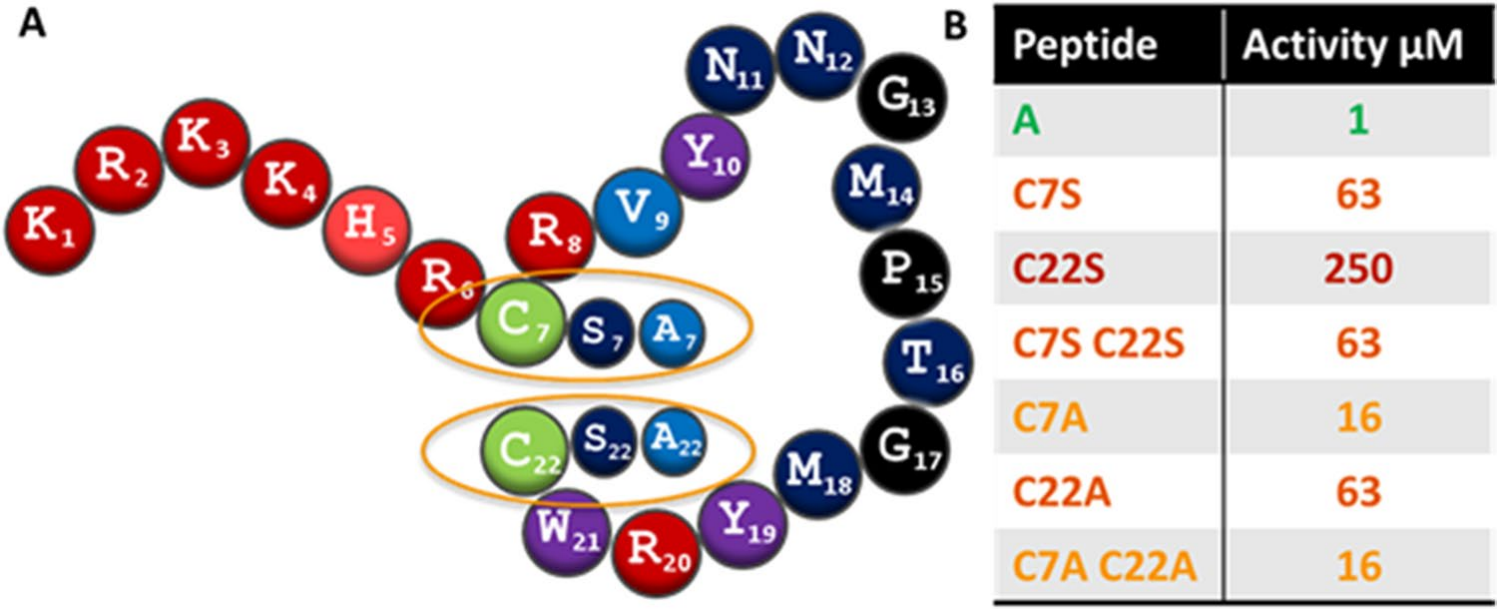
(**A**) Cys7 and Cys22 were substituted individually and in tandem with serine and alanine to give bactofencin variants C7S, C22S, C7S-C22S, C7A, C22A, C7A-C22A. Activity (µM) of bactofencin A compared to bactofencin cysteine variants C7S, C22S, C7S-C22S, C7A, C22A and C7A-C22A is presented in (**B**).

To assess the contribution of the cysteines to the activity of bactofencin A, Cys7 and Cys22 were replaced with serine. The results (Fig. 2) show that replacing Cys7 with serine results in a 63 fold reduction in activity and replacing Cys22 caused an even more detrimental 250 fold reduction in activity. Notably, replacing both Cys7 and Cys22 with a serine residue resulted in activity comparable to the C7S change alone. Replacing individual cysteine residues with alanine had less of a negative effect than serine substitution as a C7A change reduces activity 16 fold and C22A is reduced 63 fold. The C7A-C22A variant is, like the serine equivalent, comparable with the single C7A variant being 16 fold reduced. Overall, it was apparent that substituting Cys22 with either serine or alanine resulted in peptides with lower activity than those generated containing Cys7-Cys22 substitutions.

### The role of the N terminal positively charged tail

**Figure 3:**
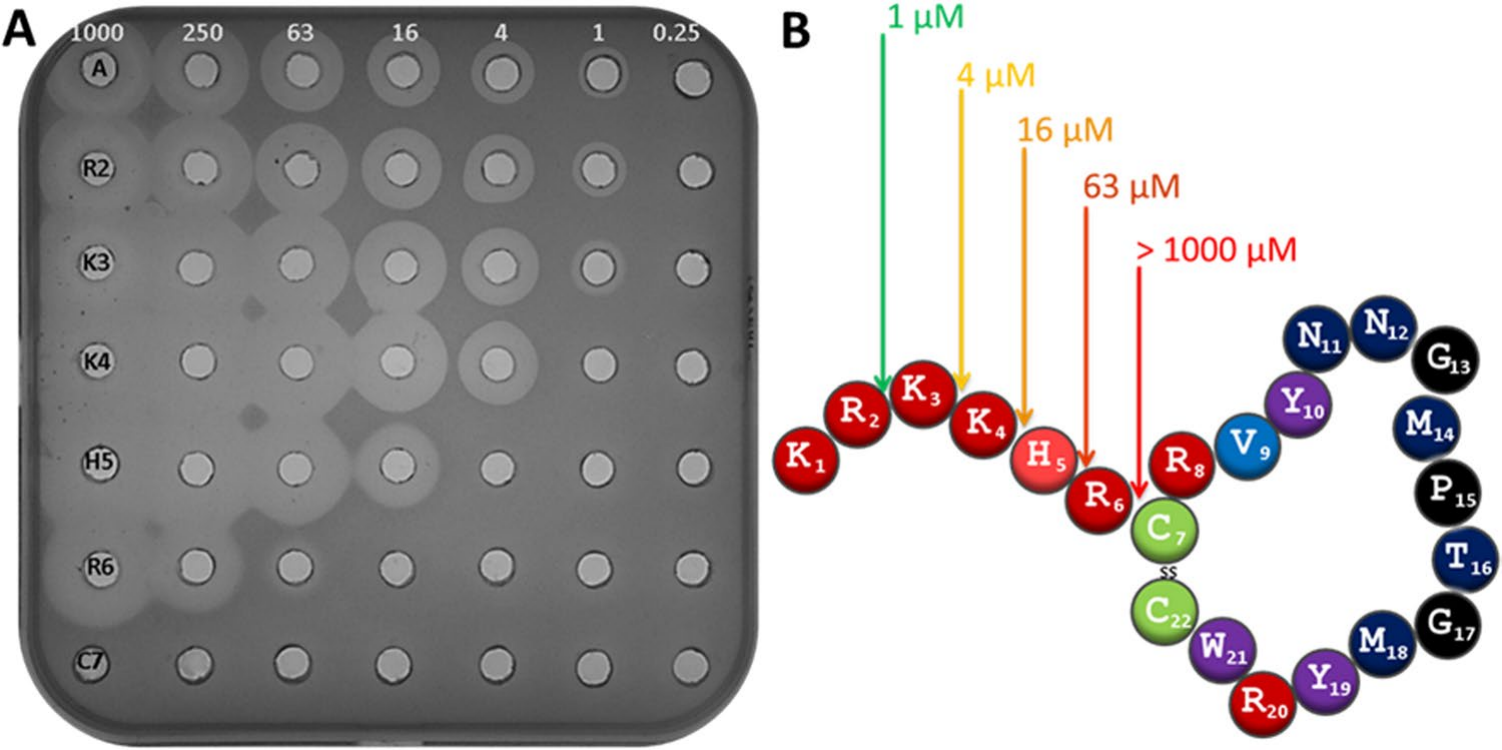
(**A**) Activity (µM) of bactofencin A and deletion variants, R2-C22, K3-C22, K4-C22, H5-C22, R6-C22 and C7-C22 against *S*. *aureus* DPC5246. (**B**) Activity (µM) of bactofencin A and deletion variants against *S*. *aureus* DPC5246. Activity is colour coded with green being most active and red inactive.

Bactofencin is characterized by a cationic N terminal, KRKKHR, where 5 out of 6 amino acids are positively charged at neutral pH. To determine how this feature contributes to the activity of the peptide, a series of deletion variants were synthesized including R2-C22, K3-C22, K4-C22, H5-C22, R6-C22 and C7-C22. The specific activity of each peptide against *S*. *aureus* was determined and reveals that deleting the first two amino acids has no effect on activity. The further exclusion of Lys3 (K4-C22), Lys4 (H5-C22) and His5 (R6-C22) resulted in sequential 4 fold reductions in activity resulting in MICs of 4 µM, 16 µM and 63 µM, respectively. Deleting the entire positively charged N terminal region resulted in a looped peptide for which no activity could be detected (MIC >1000 µM; Fig. 3).

### Alanine Scanning

**Figure 4:**
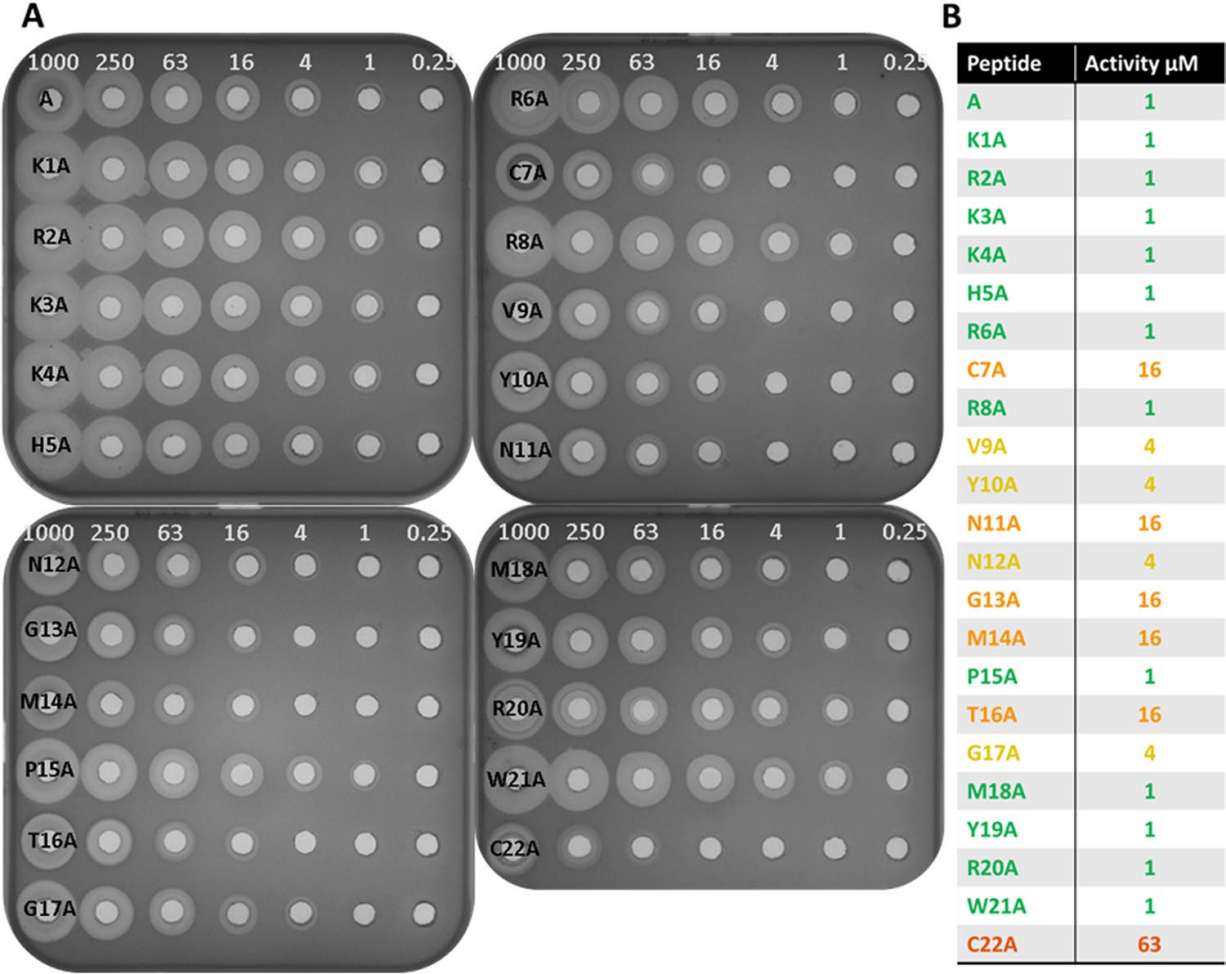
(**A**) Serial dilutions of each alanine variant plated on *S*. *aureus* DPC5246 indicator plates and (**B**) shows activity (µM) of bactofencin alanine variants against *S*. *aureus* DPC5246. Activity is colour coded with green being most active and red least active.

A series of alanine scanning variants, where each individual amino acid was replaced by alanine, were synthesized and their activity assessed. The results show that changing individual amino acids to alanine within the N terminal region had no effect on activity. This was also the case for the R8A, P15A, M18A, Y19A, R20A and W21A containing peptides. As noted previously, the C7A and C22A variants were 16 fold and 63 fold less active, respectively. Substitutions between V9A and G17A show reduced activity with V9A, Y10A, N12A and G17A being 4 fold less active and N11A, G13A, M14A and T16A being 16 fold less active (Fig. 4), thereby highlighting that this region is highly important for the antimicrobial activity of the peptide.

### D-amino acid substitution of the loop

**Figure 5:**
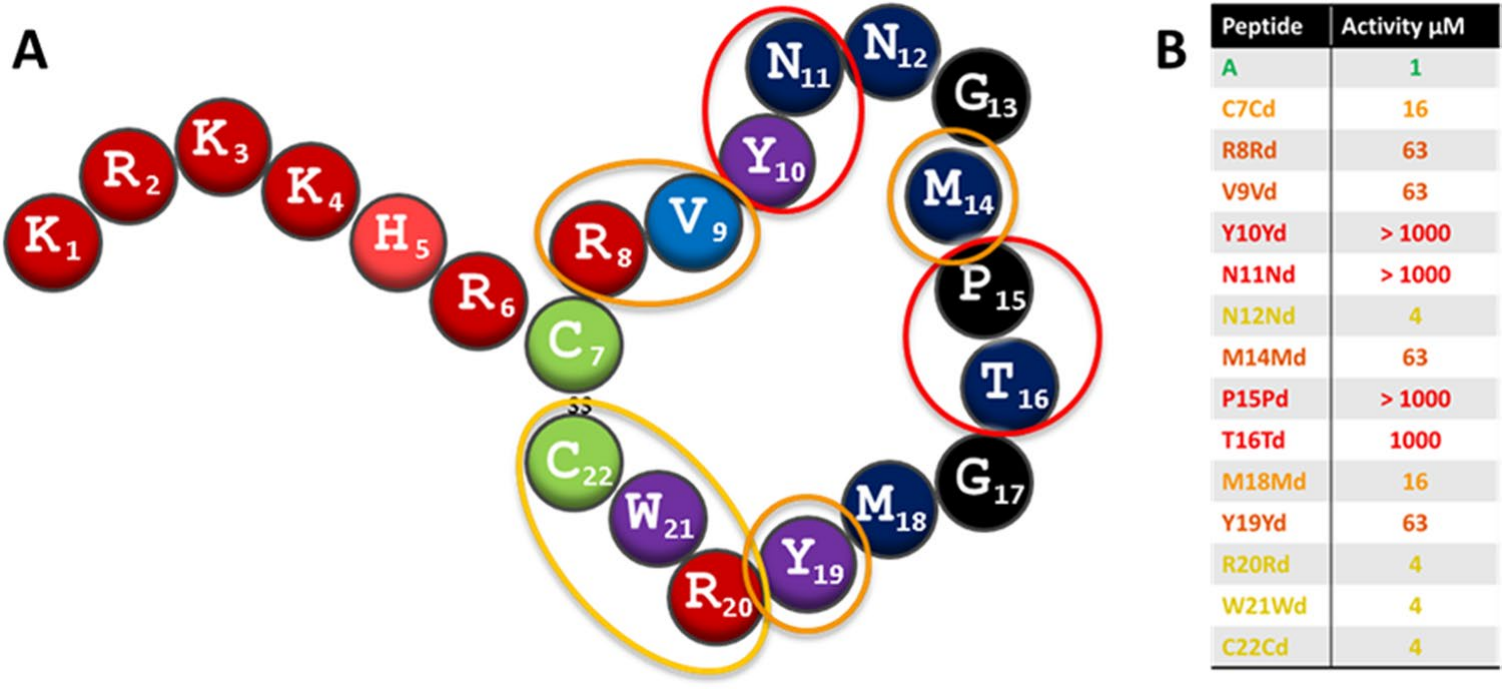
Activity (µM) of bactofencin D-variants C7Cd-C22Cd against *S*. *aureus* DPC5246. Activity is colour coded with green being most active and red inactive. Amino acids most affected by D-substitution are circled in (**A**) and activities presented in (**B**).

The generation of peptides containing D-amino acids provides an insight into the importance of chirality across the whole peptide or within specific regions. As preliminary experiments showed that an all D-variant with every amino acid changed was inactive (MIC50 >20 µM; data not shown), a series of D-amino acid variants from C7-C22 were synthesized. The D-amino acid substitutions (Fig. 5) were all found to be detrimental to activity – even more so than the equivalent alanine substitutions (with the exception of C7 and N12 which are equally detrimental at 16 and 4 μM, respectively). In the case of C22, the Ala substitution (MIC = 63 µM) is much less active than the C22Cd equivalent (MIC = 4 µM). As for the alanine-containing variants, the substitution of amino acids within the R8-Y19 region of the peptide has a particularly deleterious impact as bactofencin R8Rd, V9Vd, M14Md and Y19Yd are 63 fold less active than wild type and Y10Yd, N11Nd, P15Pd and T16Td are inactive at 1000 µM, the highest concentration tested.

### Comparison of bactofencin A with bactofencin R8Q (plantaricin ST31), a potential natural variant

**Figure 6:**
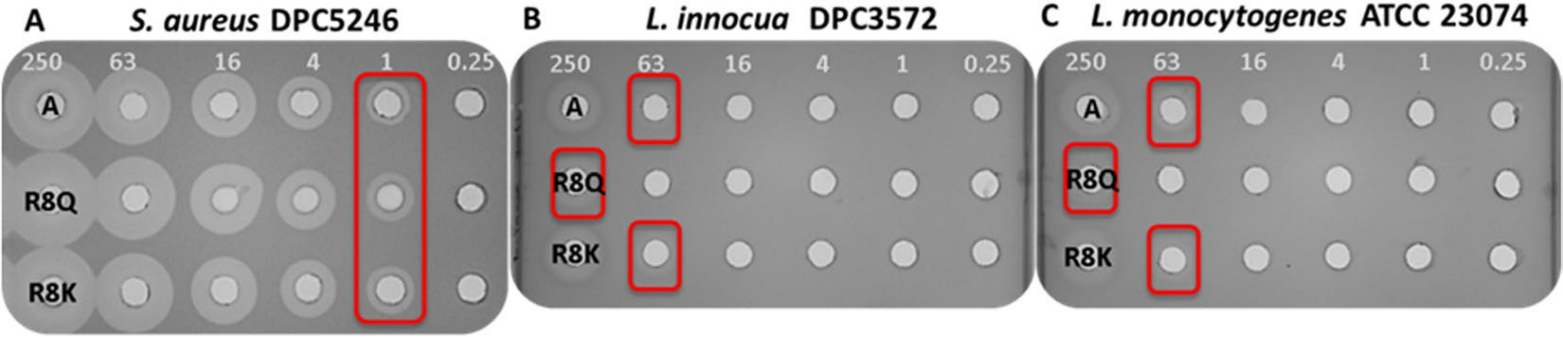
Activity (µM) of bactofencin A (**A**) bactofencin R8Q (plantaricin ST 1) and bactofencin R8K against *S*. *aureus* DPC5246 (**A**) *L*. *innocua* DPC3572 (**B**) and *L*. *monocytogenes* ATCC 23074 (**C**).

Todorov *et al*. (1999) previously described plantaricin ST31, a bacteriocin with an amino acid sequence determined to be KRKKHRXQVYNNGMPTGMYR, produced by a sourdough isolate *Lactobacillus plantarum* ST31, with a reported mass of 2755 + /− 0.3 Da^20^. Substitution of Cys for X results in a peptide with a mass of 2468 Da. However if tryptophan and cysteine, the C terminal amino acids of bactofencin A are included, a mass of 2757 Da is obtained and subsequent oxidation of cysteines gives the published mass, 2755 Da. This suggests that plantaricin ST31 is likely to be a variant of bactofencin A with an arginine to glutamine change at position 8. Although plantaricin ST31 was reported as inactive against *Listeria* spp., bactofencin A is active against *Listeria* at high concentrations. For this reason, it was decided to directly compare the activity of bactofencin R8Q (i.e. plantaricin ST31) and bactofencin A against *S*. *aureus* DPC5246, *L*. *innocua* DPC3572 and *L*. *monocytogenes* ATCC 23074. Although bactofencin A and bactofencin R8Q (plantaricin ST31) were equally active at 1 µM against *S*. *aureus* DPC5246, it was established that bactofencin A is indeed more active (63 µM) than bactofencin R8Q (plantaricin ST31; 250 µM) against *L*. *innocua* DPC3572 and *L*. *monocytogenes* ATCC 23074. The reduced activity of R8Q was not evident after a substitution that retained the charge at position 8, i.e. R8K (Fig. 6).

## Discussion

There is an urgent need for new antimicrobials to combat infection and bactofencin is a clear candidate. Although it is produced in small amounts by the producing strain, the lack of post translational modifications makes it very accessible to synthesis strategies. With this in mind, we generated a bank of synthetic variants which included a number of amino acid substitution and deletion variants. Preliminary experiments showed that the disulfide bond forms naturally over time and that synthetic bactofencin with an intact disulfide is as active as the native peptide making this a valid approach (Fig. 1). The synthetic peptide with reduced cysteines was also found to be as active as natural bactofencin suggesting that the disulfide bond is not essential for full activity (Fig. 1).

Substituting either cysteine with serine resulted in peptide variants with lower activity than alanine equivalents proving that serine is not a good substitute for cysteine in bactofencin A (Fig. 2). Cysteines and serines differ in both chemical and physical properties as cysteines are often found in the interior of a molecule especially when involved in disulfide bond formation while comparatively hydrophilic serines are typically exposed^21,22^. Alanine, however, is hydrophobic so it is possible that hydrophobic interactions between C7A and C22A are stronger than serine equivalents and so can better maintain structural conformation. This was seen when Cys9 and Cys14 residues of the Class IIa leucocin A were replaced with hydrophobic equivalents^23^. It appears that single C22 changes to either serine or alanine have a greater negative impact on activity than the corresponding C7 and C7-C22 changes. The possibility here is that substituting C22 alone with serine or alanine may introduce steric hindrance, thus preventing any semblance of loop conformation resulting in a less active peptide. Disulfides have a very distinct role in stabilizing protein structure^22^ and the results of this study suggest that the presence of both cysteines play a key role in maintaining peptide structure and are required for full activity of bactofencin A.

Sequential deletion of the positively charged N terminal from Lys3 to Arg6 resulted in a series of variants with decreasing activity against *S*. *aureus* DPC5246, while removal of the entire N terminal region prior to the loop resulted in an inactive peptide (Fig. 3). Interestingly, the C7-C22 variant contained an intact disulfide bond as determined by MALDI TOF MS. The N terminal, KRKKHR, with a charge of + 5 at neutral pH is unique among known Class II bacteriocin sequences and invites conjecture as to its function. Lysine and arginine play an important role in the interaction with negatively charged phospholipid membranes. Indeed, arginine is more effective than lysine in this regard as it forms more extensive H bonding, thereby stabilizing arginine-phosphate clusters enabling enhanced interfacial binding leading to membrane disruptions^24^. In addition, a high positive charge allows bacteriocins and AMPs to insert further into membranes^25^. It may be that KRKKHR plays a role in binding to anionic lipids in cell membranes and that a charge of at least +3 at the N terminus is required for full activity, given that variants with a lower charge were significantly less active (Fig. 3). The highly positive charge of bactofencin A may also have played a role in the evolution of the unique bacteriocin immunity associated with the producer, *L*. *salivarius* DPC6502. In this respect, immunity is mediated through a homologue of *DltB* a protein which results in the reduction in the charge of teichoic acids in the cell wall. Thus the mechanism mediating immunity could be through reducing the affinity of the positively charged bactofencin A to the producer surface^10^.

Alanine scanning mutagenesis approaches have been successfully used to study the lantibiotic, lacticin 3147^26^ and the Class IIa bacteriocin, durancin GL^27^. In bactofencin A, replacing the amino acids of the N terminal with alanine did not lead to a reduction of activity when compared to the native peptide. This correlates with the results from the deletion experiment as it is expected that the loss of a single positive charge would not adversely affect activity. Changing Cys7 to alanine does have an effect but this is expected due to possible structural changes in the peptide as speculated earlier. Changes to Arg8 and the C terminal side of the loop were also well tolerated as R8A, P15A, M18A, Y19A, R20A and W21A are as active as bactofencin A. However, when residues between Val9 and Gly17 are changed to alanine activity is significantly reduced, suggesting that this part of the loop makes an important contribution to activity (Fig. 4).

D-amino acid substitutions were used to investigate the importance of stereochemistry for target interaction as introduction of D-amino acids typically disrupts the helicity of AMPs^28^. The retention of significant levels of activity in an all D variant of AMPs suggests the natural peptide functions by interacting with the lipid membrane, rather than a specific receptor, whereas a significant reduction in activity among such variants suggests that a stereospecific target, such as a membrane receptor, is involved in activity^29^. The latter proved to be the case for bactofencin A. Substituting the amino acids of the loop for D equivalents was detrimental to activity in every case but most particularly in peptides with substitutions between Arg8 and Tyr19. Indeed, Y10Yd, N11Nd, P15Pd and T16Td were totally inactive at 1000 µM further suggesting a chiral interaction between bactofencin A and a specific receptor or that the introduction of a D residue disrupted structural conformation within this region. The inactivity of the P15Pd peptide is particularly notable and is in stark contrast to the activity observed with the P15A variant, suggesting that kinking the molecule in the opposite direction leads to detrimental structural changes.

Taken together, the results from the N-Terminal deletion, alanine scanning and D amino acid variant studies make it tempting to suggest that bactofencin A interacts with the cell membrane through initial electrostatic interaction with the N terminal and then disrupts the cell through binding with a putative receptor to amino acids in the interior of the loop.

The existence of an apparent natural variant of bactofencin A is interesting and provides an opportunity to compare the activities of the two peptides, especially in light of the absence of activity in cell free supernatants of plantaricin ST31-producing *L plantarum* ST31 against *L*. *innocua* and *L*. *monocytogenes*^20^. This natural substitution did indeed result in reduced activity against *Listeria* as evidenced by our studies with bactofencin R8Q (plantaricin ST31). Substituting glutamine for lysine, the other positively charged amino acid restored activity suggesting that a positive charge is necessary in this position for activity against *Listeria*.

None of the bactofencin A variants investigated in this study resulted in enhanced anti-staphylococcal activity with respect to the native bactofencin suggesting that wild type bactofencin A has close to maximal antimicrobial activity. However, the reduction in activity against *Listeria* spp. due to an R8Q change and its restoration with an R8K change suggests that there is potential to change the spectrum of activity of bacteriocins. Ultimately, it will now be possible to build on this blueprint to further investigate the fundamental biology underlying the activity of bactofencin and, in turn, enhance the spectrum and activity of this cationic bacteriocin.
